# The methodology of resistance training is crucial for improving short-medium distance front crawl performance in competitive swimmers: a systematic review and meta-analysis

**DOI:** 10.3389/fphys.2024.1406518

**Published:** 2024-07-26

**Authors:** Guoli Jin, Yangqin Jin, Haoyang Zhang, Xueying Fu, Yong Yang, Shu-Cheng Lin

**Affiliations:** ^1^ School of Physical Education and Sport, Quanzhou Normal University, Quanzhou, China; ^2^ Physical Training Research Laboratory, Belarusian National Technical University, Minsk, Belarus; ^3^ Physical Training Research Laboratory, School of Physical Education and Sport, Henan University, Kaifeng, China; ^4^ Physical Training Research Laboratory, School of Physical Education and Sport, Chaohu University, Hefei, China; ^5^ Department of Sport, Leisure and Health Management, Tainan University of Technology, Tainan, Taiwan

**Keywords:** resistance training, competitive swimmers, swimming performance, stroke rate, stroke length

## Abstract

**Background:**

Resistance training is often a part of the routine training regimen for competitive swimmers. However, due to the variety of resistance training methodology, the results can be inconsistent and sometimes unsatisfactory. Clear recommendations are still lacking at present.

**Aims:**

1) Quantify the impact of resistance training on swimmers’ upper limb maximum strength, front crawl performance and key technical parameters; 2) Find out the key technical parameters for improving front crawl performance—stroke rate or length; 3) Through subgroup analysis determine the best methodology of resistance training to enhance the front crawl.

**Methods:**

Systematic search in the PubMed, Embase, and Web of Science databases. Meta-analyses using the inverse-variance are performed to compare swimmers’ upper limb maximum strength, front crawl performance and key technical parameters in resistance training and habitual aquatic training. A subgroup analysis was performed to examine whether the results were affected by the methodology of resistance training.

**Results:**

Thirteen studies (267 competitive swimmers) met the inclusion criteria. The results of meta-analysis showed that resistance training significantly improved upper limbs maximum muscle strength, and 25, 50, 100, and 200 m front crawl performance in competitive swimmers. And improvements in swimming performance may simply resulted from resistance training increasing stroke rate rather than stroke length. In addition, the results of subgroup analysis showed that only concurrent resistance training (CRT) and power training (PT) ultimately improved swimming performance by increasing the maximum muscle strength of the upper limbs.

**Conclusion:**

Resistance training significantly enhances competitive swimmers’ upper limb strength and front crawl performance across various distances. The improvement in performance is likely attributed to an increased stroke rate rather than stroke length. In addition, CRT and PT are particularly effective, indicating the importance of selecting the appropriate methodology of resistance training for optimal swimming performance enhancement.

**Systematic review registration:**

https://doi.org/10.17605/OSF.IO/3JEGW.

## 1 Introduction

Resistance training can be defined as an exercise modality that involves muscle contractions against external resistance to enhance muscle strength (Fleck and Kraemer, 2014), which is an essential physical training program for competitive athletes (Granacher et al., 2016). Studies suggest that resistance training has a moderate positive effect on Olympic timing events (Girold et al., 2007). One explanation for how increased muscle strength might improve performance in Olympic timing events is that, as strength increases, the relative load on the working muscles decreases, potentially optimizing the activation of motor neurons (Sunde et al., 2010), thus reducing the energy cost of movement. Moreover, the reduction in relative load can also decrease the rate of local muscle fatigue, enabling athletes to maintain optimal movement speed over a longer duration (Girold et al., 2007), leading to faster competition times.

Swimming performance (time taken to test the corresponding distance) is influenced by a complex interaction of physiological, biomechanical and technical factors (Barbosa et al., 2010; Costa et al., 2012). Swimming speed is the product of stroke rate and stroke length (Wakayoshi et al., 2005). And independently increasing stroke rate (Girold et al., 2006) or stroke length (Wakayoshi et al., 1995) had been found to improve swimming performance (time taken to test the corresponding distance). Additionally, resistance training is a safe and feasible method for competitive swimmers to increase their muscle strength, and well-developed muscle strength plays an important role in achieving short-medium distance swimming performance in adolescent and young adult swimmers (Amara et al., 2021b; Lopes et al., 2021). Previous research had shown that 30 sessions of dry-land resistance training over 3 weeks significantly increased stroke rate but not stroke length, and ultimately improved 50 m front crawl performance (Girold et al., 2006). The research results of Khiyami et al. (2022) showed that dry-land resistance training with a total of 18 sessions over a 6-week period significantly improved the 50 m front crawl performance of competitive swimmers mainly from the length of stroke rather than stroke rate. Recent research results showed that the reason why dry-land resistance training for a total of 24 sessions over 8 weeks improved the performance of the 100 m butterfly was due to the significant improvement in stroke rate and stroke rate (Amara et al., 2023). Clearly, there is still controversy on how resistance training improves key technical parameters in swimming and ultimately enhances swimming performance.

There are many forms of resistance training available for swimmers, and the effects of improving sports performance vary. For example: The research results of Augusto C. Barbosa et al. (2020) showed that a total of 12 times of aquatic resistance training with a hand paddles for 4 weeks did not significantly improve the 50 m front crawl performance of competitive swimmers. Even if the number of training sessions was increased, 30 sessions of aquatic resistance training (providing fixed push off points in the water for swimming) over a 10-week period did not significantly improve the performance of competitive swimmers in the short-medium distance (50, 100, and 200 m) front crawl compared to habitual aquatic training (Toussaint and Vervoorn, 1990). However, similar training doses (12 sessions over 4 weeks) of dry-land resistance training and dry-land high speed resistance training significantly improved the performance of competitive swimmers in the 50 m front crawl (Naczk et al., 2017; Norberto et al., 2023). In recent years, concurrent resistance training (combined aquatic and dry-land resistance training) attracted attention, and the results showed that had a significant increase in the performance of competitive swimmers (Amara et al., 2021a; Amara et al., 2022). Therefore, there is still a lack of clear recommendations on how to select a significantly effective form of resistance training.

In summary, it is still unclear how to choose resistance training methodology and how resistance training can improve key technical parameters of swimming and ultimately improve swimming performance. Therefore, the purposes of this systematic review and meta-analysis are to: 1) Quantitatively analyze the effects of resistance training methods on maximum muscle strength of upper limbs, short-medium distance front crawl swimming performance and key technical parameters of competitive swimmers; 2) To explore what is the key parameter for resistance training to improve the short-medium distance front crawl performance of competitive swimmers, stroke rate or stroke length? 3) To quantify the optimal methodology of resistance training for improving front crawl performance in competitive swimming through subgroup analysis.

## 2 Methods

Our systematic review and meta-analysis were carried out in strict compliance with the Preferred Reporting Items for Systematic Reviews and Meta-Analyses (PRISMA) guidelines (Liberati et al., 2009) (Supplementary Appendix S1). This review was prospectively registered with Open Science Framework (https://doi.org/10.17605/OSF.IO/3JEGW). No amendments to the protocol occurred after registration, and no protocol was prepared.

### 2.1 Literature search

We performed a systematic search in the PubMed, Embase, and Web of Science databases from the date of their inception to 18 February 2024, with no language restrictions. When searching for eligible articles, we used the search terms (“strength training” OR “resistance training” OR “weight training” OR “power training” OR “plyometric training” OR “complex training” OR “weight-bearing exercise”) AND (“swimming athlete” OR “athlete” OR “swimmer” OR “competitive swimmers”) (Supplementary Appendix S2). The reference lists of the included studies and previous reviews were screened for additional studies. GLJ and JYW screened all search results to exclude duplicates. The titles and abstracts of the remaining studies were independently screened by GLJ and JYW based on inclusion and exclusion criteria. Full texts that met these criteria were independently screened by GLJ and JYW, and all disagreements were decided by YY.

### 2.2 Inclusion and exclusion criteria

In alignment with the PICOS framework (Hutton et al., 2015), our inclusion criteria were meticulously defined as follows: (a) Participants consisted of competitive swimmers; (b) The intervention included resistance training along with its advanced variations; (c) The comparison was made against habitual aquatic training routines; (d) Outcomes measured encompassed pre- and post-intervention maximum muscle strength of upper limbs, performance in the 25–200 m front crawl (seconds), maximum velocity (meters/second), stroke rate (cycles/second), and stroke length (meters/cycle); (e) The study design was restricted to published Randomized Controlled Trials (RCTs), whether individually designed, cluster-designed, or the initial phase of crossover studies. We excluded research focusing on the acute effects of a singular session on competitive swimmers and studies that lacked clear descriptions of the resistance training employed. Studies were also excluded if they did not provide means and standard deviations in their results or if the authors did not respond to our data inquiries.

### 2.3 Coding of studies

Upon reviewing the titles and abstracts, we conducted a comprehensive assessment of the full texts for all pertinent articles. During this phase, two authors (GLJ and JYW) meticulously extracted data on (1) characteristics of the participants, such as sample size, age, and gender; (2) detailed descriptions of the interventions, including methodology of training; (3) specifics of the training variables; and (4) the main results of the study. In instances where raw data were incomplete, we reached out to the corresponding authors for clarification. Studies were excluded if the authors were unresponsive. GLJ and JYW independently evaluated all studies, utilizing the extracted data as their guide. Any discrepancies regarding the inclusion of a study were resolved through consultation with YY. The data from the studies that met our inclusion criteria are summarized in Table 1.

**TABLE 1:** Characteristics of the studies and subjects included in the review.

Study	Level	Mean age	Sample size (male/female)	Height	Body mass (kg)	Swimming experience (years)	Intervention details
Amara et al. (2022)	National competitive swimmers	CRT: 16.5 ± 0.3 HAT: 16.1 ± 0.3	CRT: 11(11/0) HAT: 11(11/0)	CRT: 174 ± 9.8 HAT: 175 ± 9.7	CRT: 72.7 ± 5.3 HAT: 73.6 ± 5.25	>5	a: aquatic resistance training with a small water parachute, 2–3 sets × 4–6 reps × 15–25 m with 60–90 s and 5 min of rest between repetitions and sets, low and high intensity interval, 4 times/week, 9 weeks b: lower body strength training, 3 sets, 60%–80% 1RM, 6–8 reps back squat, CMJ and CMJ box exercises consisted of 6–8 sets with 6–10 repetitions, 2 times/week, 9 weeks
Amara et al. (2021a)	National competitive swimmers	CRT: 16.5 ± 0.30 HAT: 16.1 ± 0.32	CRT: 11(11/0) HAT: 11(11/0)	CRT: 174 ± 9.8 HAT: 175 ± 9.7	CRT: 72.7 ± 5.3 HAT: 73.6 ± 5.25	CRT: 6.86 ± 0.33 HAT: 4.78 ± 0.34	a: resistance training in water with a small water parachute or hand paddles, On Monday and Thursday, the swimmers performed 3 sets × 4 reps × 15 m; On Tuesday, the swimmers performed 2 sets × 4 reps × 25 m and, on Friday, underwent 2 sets × 4 reps × 25 m, 90 s and 5 min of rest between reps and sets, low and high intensity interval, 4 times/week, 9 weeks b: resistance training in the dry land, 2–5 kg ball throw and 60%–80%1RM bench press, 3–6 sets, 6–12 reps, 2 times/week, 9 weeks
Augusto C. Barbosa et al. (2020)	Competitive swimmers	ART: 21.8 ± 1.9 HAT: 22.4 ± 2.3	ART: 10(5/5) HAT: 10(5/5)	ART: 1.70 ± 0.11 HAT: 1.72 ± 0.08	ART: 65.5 ± 12.8 HAT: 70.5 ± 10.5	>2	low, moderate and high-intensity zones, swimming with hand paddles, 10 × 10 strokes all-out, 1-min rest, 3 times/week, 4 weeks
Gourgoulis et al. (2019)	National competitive swimmers	13.08 ± 6.9	ART: 6(0/6) HAT: 6(0/6)	158 ± 5	48.3 ± 6.9	3.92 ± 0.9	a: swimming with water parachute, on Mondays and Thursdays, 3 sets of 6 × 15 m with 60 s rest between reps, and 5 min rest between sets; on Mondays and Thursdays, 2 sets of 4 × 25 m with 90 s rest between reps, and 5 min rest between sets, 4 times/week, 11 weeks b: swimming without water parachute, on Wednesdays and Saturdays, 2 times/week, 11 weeks
Khiyami et al. (2022)	Competitive swimmers	RT: 13 ± 2 HAT: 13.11 ± 2.6	RT: 9(9/0) HAT: 9(9/0)	RT: 158.8 ± 17.3 HAT: 160.4 ± 11.9	RT: 48.3 ± 14.2 HAT: 49.1 ± 11.3	RT: 2.8 ± 0.4 HAT: 2.9 ± 0.7	a: core strength training 1 min between exercises, 3 times/week, 6 weeks b: habitual aquatic training
Naczk et al. (2017)	National competitive swimmers	15.8 ± 0.4	RT: 7(5/2) HAT: 7(5/2)	179 ± 7	69 ± 8	≥6	a: dry-land inertial training, strength training using the inertial training measurement system, 3 times/week, 4 weeks b: habitual aquatic training
Norberto et al. (2023)	Competitive swimmers	15.6 ± 2.1	29(16/13)	164.7 ± 5.6	58.8 ± 4.4	≥2	PT: a: power (plyometric bench-press, barbell squat clean and press, reactive pull-over and barbell jump squat) and strength (bench triceps dips, pull-up), 3 times/week, 4 weeks b: habitual aquatic training RT: performed training on conventional cable and stack training equipment containing only strength exercises (machine biceps preacher, bench-press machine, wide grip lat pulldown, leg-press, triceps cable rope push, machine calf raises), 3 times/week, 4 weeks b: habitual aquatic training
Pinos et al. (2021)	Competitive swimmers	16.5 ± 0.9	RT:7(4/3) HAT:7(4/3)	176.2 ± 8.3	64.9 ± 7.3	NA	a: strength training using the swimming ergometer, 3 sets of 3 exercises 80%–90% 1RM, 2 times/week, 4 weeks b: and habitual aquatic training
Sammoud et al. (2021)	Competitive swimmers	PT: 10.01 ± 0.57 HAT: 10.50 ± 0.28	PT: 12(0/12) HAT: 10(0/10)	PT: 146.90 ± 7.62 HAT: 143.60 ± 5.05	PT: 36.39 ± 6.32 HAT: 38.41 ± 9.42	2 ± 1.4	a: power training, jump exercises, 8–12 sets with 6–10 reps, 2 times/week, 8 weeks b: habitual aquatic training
Sammoud et al. (2019)	Competitive swimmers	PT:10.3 ± 0.4 HAT:10.5 ± 0.4	PT:14(14/0) HAT:12(12/0)	PT:143 ± 8 HAT:146 ± 7	PT:36.2 ± 8.4 HAT:38.2 ± 5.9	2 ± 1.6	b: power training, jump exercises, 8–12 sets with 6–10 reps, 2 times/week, 8 weeks b: habitual aquatic training
Girold et al. (2012)	Regional/national competitive swimmers	RT: 21.1 ± 1.4 HAT: 24.2 ± 4.6	RT: 8(4/4) HAT: 8(4/4)	RT: 176 ± 8 HAT: 175 ± 7	RT: 69.1 ± 6.5 HAT: 69.3 ± 7.4	10 × 2 h wk −1	a: pull-ups and draws with pulleys, 80%–90% 1RM, 3 sets of 3 exercises with 2 min of rest between each set, 6 reps, 3 times/week, 4 weeks b: habitual aquatic training
Girold et al. (2006)	Regional/national competitive swimmers	ART: 16.5 ± 2 HAT: 17 ± 3	37(16/21)	ART:170 ± 7 HAT:168 ± 9	ART: 58 ± 9.5 HAT: 62 ± 11	NA	aquatic resistance training, wearing an elastic band around your waist to increase resistance while swimming, and habitual aquatic training, 10 times/week, 3 weeks
Toussaint and Vervoorn (1990)	Competitive swimmers (competed at Dutch nationals)	ART: 18.5 ± 3.3 HAT: 18.4 ± 2.1	ART: 11(8/3) HAT: 11(8/3)	ART: 178 ± 8 HAT: 179 ± 7	ART: 69.2 ± 7.8 HAT: 72.3 ± 8	NA	providing fixed push off points in the water for swimming, 3 times/week, 10 weeks

**habitual aquatic training:** ART, aquatic resistance training, CRT, concurrent resistance training RT, resistance training, HAT, habitual aquatic training, PT, power training, NA, non-available.

### 2.4 Assessment of risk of bias

The risk of bias for each individual study was assessed independently by GLJ and JYW using the using the Cochrane Risk of Bias version 2 tool (RoB2) (Sterne et al., 2019), including five domains: randomisation process; deviations from intended interventions; missing outcome data; outcome measurement; and selection of reported results. For cluster randomized controlled trials, the RoB 2.0 tool uses an additional domain to assess risk of bias due to the timing of identifying and recruiting participants (Eldridge et al., 2016), in addition to the five domains above. Each area was assessed as (1) high risk, (2) low risk and (3) some concern. For each study, if all domains showed low risk, the overall risk of bias was low; if any of the above domains showed high risk, or the assessment results of multiple domains showed some concern, the overall risk of bias was high; otherwise, Overall risk of bias was low. Disagreements were resolved by consensus among reviewers or in consultation with a third reviewer.

### 2.5 Quality of the evidence

Two independent reviewer (GLJ and JYW) also assessed the strength of the current evidence for each outcome and subgroup using the GRADE method (Guyatt et al., 2008). In our review, the evidence started with high quality and was downgraded for each of the flowing issues: (1) publication bias (at least 10 studies, Egger test *p*-value < 0.05) (Ioannidis and Trikalinos, 2007); (2) imprecision when fewer than 400 subjects were included in the meta-analysis (Mueller et al., 2007); (3) when more than 25% of the studies from high risk of bias were included (Mascarenhas et al., 2021); (4) I^2^ > 50% in the heterogeneity (Higgins and Green, 2008). We choose to summarise judgments across domains using the 4 levels of confidence of the GRADE approach: very low, low, moderate, or high (Brozek et al., 2009).

### 2.6 Statistical analyses

To determine the effectiveness of resistance training on performance in competitive swimmers. Missing standard deviations (SDs): When standard errors (SEs) instead of SDs were presented, the former was converted to SDs: (SD = SE*√n). If both were missing, we estimated the SD from credible intervals (CrIs), t values, or *p* values as described in Section 7.7.3 of the Cochrane Handbook for Systematic Reviews (Higgins and Green, 2011). The amount of baseline and post change between the experimental group and control group were calculated by the following formula: Meanchange=Meanpost − Meanbaseline; SDchange=SQUAT SDbaseline^2+SDpost^2 − 2*R*SDbaseline*SDpost, where R is a constant (R = 0.5) (McKenzie and Brennan, 2019). The effect sizes were calculated as mean differences (MD) for continuous outcomes. Due to maximum muscle strength of upper limbs was assessed by different test, we chose the standard mean difference (SMD) to assess effect size. To evaluate the reliability of our estimates, we utilized 95% CrIs. We used the I^2^ test and *p*-value to analyze the statistical heterogeneity between the studies, and when I^2^ > 50%, it meant that there was substantial heterogeneity, we chose a random effects model, otherwise a fixed effects model. We conducted quantitative analysis through Egger’s test for all outcomes to investigate the presence of publication bias (*p* < 0.05, indicating significant publication bias). Based on the resistance training methodology, a subgroup analysis was performed to examine whether resistance training methodology influence the efficacy of resistance training on athletic performance. Data analysis was achieved based on the R statistical environment (V.4.3.2, www.r-project.org). The measured effects were considered significant at *p* < 0.05.

## 3 Results

### 3.1 Study selection

**FIGURE 1:**
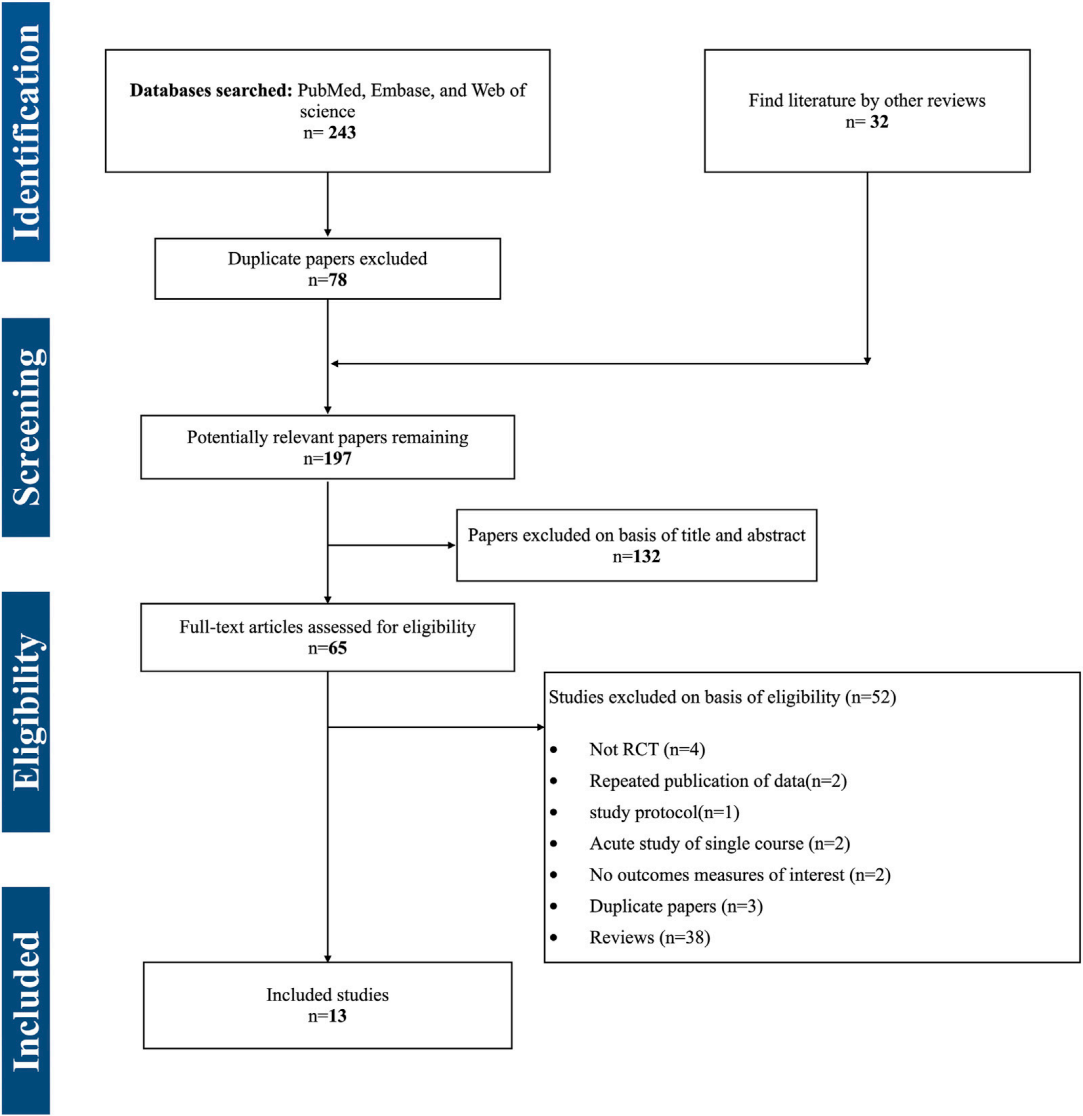
PRISMA Flow diagram of the search process for studies. RCT randomized controlled trials.

The literature search identified 243 potentially relevant studies (Figure 1). A total of 78 duplicates were removed, and a screening of the titles and abstracts excluded 132 studies. A total of 65 full-text articles were retrieved, GLJ and JYW confirmed the outcomes of interest by viewing the full text, and 13 studies (Toussaint and Vervoorn, 1990; Girold et al., 2006; Girold et al., 2012; Naczk et al., 2017; Gourgoulis et al., 2019; Sammoud et al., 2019; Barbosa et al., 2020; Amara et al., 2021a; Pinos et al., 2021; Sammoud et al., 2021; Amara et al., 2022; Khiyami et al., 2022; Norberto et al., 2023) with 267 participants (male: 168, female: 99), mean age of 16.1 years (age range 10–24 years), mean height of 151.1 cm (range 143–179 cm), mean body weight of 59.8 kg (range 36.2–73.6 kg), regional or national competitive swimmers with 2–8.7 years of training were included in the systematic review and meta-analysis (Table 1).

### 3.2 Description of the included studies

The resistance training lasted for 3–11 weeks, with total sessions of 12–54 times. Two studies in the included literature comparing concurrent resistance training (CRT) (using paddling or water parachute in the water and dry-land resistance training) with the habitual aquatic training (Amara et al., 2021a; Amara et al., 2022). Four studies were direct comparisons of aquatic resistance training with water parachute or hand paddles (ART) and the habitual aquatic training (Toussaint and Vervoorn, 1990; Girold et al., 2006; Gourgoulis et al., 2019; Barbosa et al., 2020). Four studies were direct comparisons of dry-land resistance training (DLRT) and the habitual aquatic training (Girold et al., 2012; Naczk et al., 2017; Pinos et al., 2021; Khiyami et al., 2022), 2 studies were direct comparisons of power training (PT) and the habitual aquatic training (Sammoud et al., 2019; Sammoud et al., 2021). One study involved direct comparisons of DLRT, PT, and habitual aquatic training (Norberto et al., 2023).

### 3.3 Main effects

**FIGURE 2:**
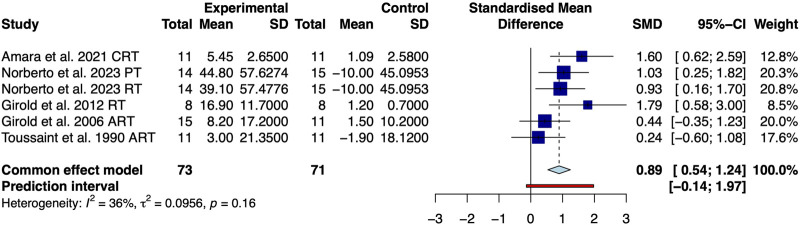
Meta-analysis of resistance training on maximal upper limb strength in competitive swimmers.

Five studies involving 129 swimmers assessed maximum muscle strength of upper limbs (Table 2). Overall, the results of low quality showed that resistance training significantly improved the maximum muscle strength of upper limbs of swimmers compared with the habitual aquatic training [SMD: 0.89, 95% CrI (0.54, 1.24), I^2^ = 36%, Figure 2].

**TABLE 2:** Pre- and post-test changes in the assessed outcome indicators for each group.

Study	Upper limbs maximum strength tests	Pool length (m)	Swimming performance	Swimming velocity	Stroke rate	Stroke length
Amara et al. (2022)	NA	50	100 m fc (s): 2.63 ± 1.5^a^ vs. −0.28 ± 1.55	NA	NA	NA
Amara et al. (2021a)	One maximum repetition of the bench press (kg): 5.45 ± 2.65 vs. 1.09 ± 2.58*	NA	25 m fc (s): 0.9 ± 0.49^a^ vs. −0.16 ± 0.6 50 m fc (s): 1.15 ± 0.92^a^ vs. −0.07 ± 0.75	Elimination of start and end effects during 25 m fc test, average velocity over 10 m (m/s): 0.16 ± 0.10^a^ vs. 0.01 ± 0.11	The time required for three consecutive stroke cycles and then the average is calculated (cycles/s): 0.16 ± 0.10^a^ vs. 0.01 ± 0.11	V/SR (m/cycle): 0.07 ± 0.06 vs. −0.04 ± 0.04
Augusto C. Barbosa et al. (2020)	NA	25	50 m fc (s): 0.00 ± 4.82 vs. −0.1 ± 4.28	Elimination of start and end effects during 25 m fc test, average velocity over 10 m (m/s): 0.00 ± 0.21 vs. 0.00 ± 0.17	^b^The time required for three consecutive stroke cycles and then the average is calculated (cycles/min): 0.4 ± 4.8 vs. 1.1 ± 4.2	V/SR (m/cycle): 0.01 ± 0.17 vs. −0.02 ± 0.14
Gourgoulis et al. (2019)	NA	25	50 m fc (s): 1.15 ± 2.05 vs. −0.07 ± 3.29 100 m fc (s): 3.98 ± 5.23 vs. −0.9 ± 7.87 200 m fc (s): 12.93 ± 8.99^a^ vs. −1.0 ± 9.12	SL^a^SR (m/s): 0.02 ± 0.05 vs. 0.00 ± 0.11	The time required for two consecutive complete right arm stroke cycles and then the average is calculated(cycles/s): 0.04 ± 0.10 vs. −0.02 ± 0.11	Stroke length was calculated as the average longitudinal displacement of the right hip over two consecutive cycles of the same arm (m/cycle): 0.03 ± 0.19 vs. 0.02 ± 0.17
Khiyami et al. (2022)	NA	50	50 m fc (s): 1.6 ± 8.3 vs. −0.1 ± 4.0	Total distance covered (50 m) divided by the time required to cover that distance (m/s): 0.00 ± 0.26 vs. 0.00 ± 0.17	^b^By taking the average of three complete cycles divided by three (cycles/min): 3.5 ± 8.1 vs. −0.7 ± 4.3	V/SR (m/cycle): 0.90 ± 0.70 vs. 0.60 ± 0.44
Naczk et al. (2017)	NA	NA	50 m fc (s): 0.22 ± 1.01 vs. −0.03 ± 1.00 50 m fc (s): 1.24 ± 2.74 vs. −0.12 ± 2.61	NA	NA	NA
Norberto et al. (2023)	Peak force was the highest force value during the 30 s of maximum effort: 44.8 ± 57.6^a^ vs. 39.1 ± 57.5^a^ vs. −10 ± 45.1	NA	50 m fc (s): 1.7 ± 4.3 vs. −1.0 ± 5.5 vs. −0.3 ± 1.94 100 m fc (s): 4.5 ± 3.2^a^ vs. −3.8 ± 4.9 vs. −0.5 ± 4.30 200 m fc (s): 8.9 ± 8.04^a^ vs. −4.3 ± 9.06 vs. −1.9 ± 8.96	NA	NA	NA
Pinos et al. (2021)	NA	50	50 m fc (s): 0.4 ± 1.4 vs. −0.4 ± 1.41	The average swim speed for the 50 m effort (m/s): 0.00 ± 0.10 vs. −0.00 ± 0.10	NA	NA
Sammoud et al. (2021)	NA	50	25 m fc (s): 1.22 ± 1.06^a^ vs. −0.15 ± 1.11 50 m fc (s): 1.7 ± 2.43 vs. 0.43 ± 2.85	NA	NA	NA
Sammoud et al. (2019)	NA	50	25 m fc (s): 0.68 ± 0.82 vs. −0.27 ± 1.31 50 m fc (s): 0.9 ± 1.6 vs. 0.10 ± 3.55	NA	NA	NA
Girold et al. (2012)	Peak torque of the arm extensors in concentric at 180/s (N/m): 16.9 ± 11.7^a^ vs. 1.2 ± 0.7	25	50 m fc (s): 0.53 ± 0.62 vs. −0.28 ± 0.91	NA	^b^The mean of the total number of cycles recorded during the 50 m was used to calculate stroke rate (cycles/min): 1.2 ± 3.6 vs. 0.6 ± 2.4	The mean of the total number of cycles recorded during the 50 m was used to calculate stroke length (m/cycle): 0.01 ± 0.11 vs. 0.02 ± 0.03
Girold et al. (2006)	Peak torque of elbow extensors at 180/s (N/m): 8.2 ± 17.2 vs. 1.5 ± 10.2	25	NA	NA	^b^The mean of the total number of cycles recorded during the 50 m was used to calculate stroke rate (cycles/min): 0.6 ± 4.8 vs. −1.2 ± 3.2	The mean of the total number of cycles recorded during the 50 m was used to calculate stroke length (m/cycle): 0.02 ± 0.19 vs. 0.06 ± 0.20
Toussaint and Vervoorn (1990)	Peak force using the MAD-device (N): 3.0 ± 21.35 vs. −1.9 ± 18.12	50	50 m fc (s): 0.6 ± 1.8 vs. −0.4 ± 1.9 100 m fc (s): 1.9 ± 3.7 vs. −1.3 ± 4.5 200 m fc (s): 2.3 ± 8.28 vs. −0.9 ± 10.57	Using the MAD-device (m/s): 0.06 ± 0.11 vs. 0.02 ± 0.12	NA	NA

NA, None-available, fc front crawl.

^a^ Significant differences compared to the habitual aquatic training.

^b^ When data is merged, the unit is converted into cycles/s.

**FIGURE 3:**
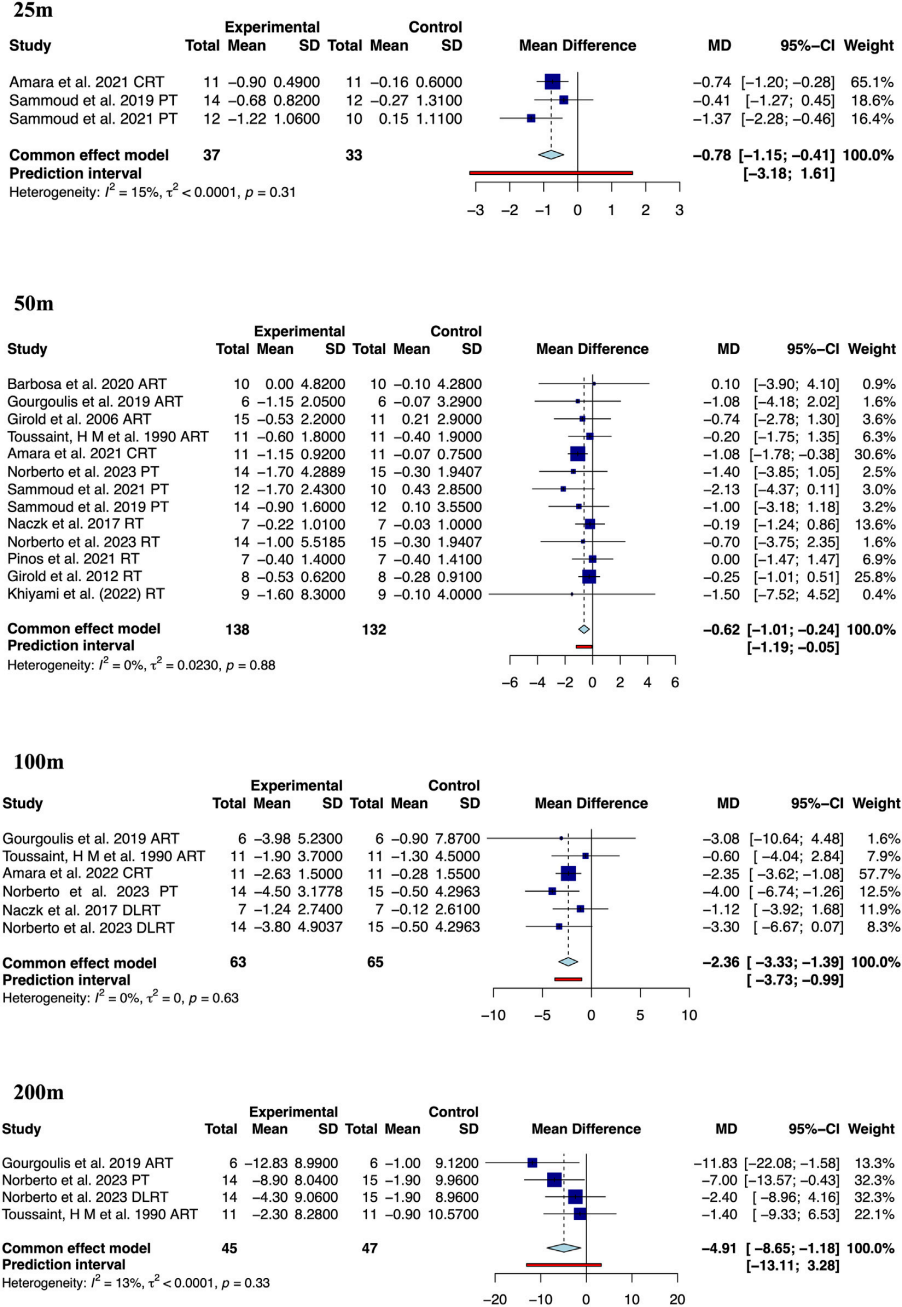
Meta-analysis of resistance training on 25–200 m front crawl in competitive swimmers.

**FIGURE 4:**
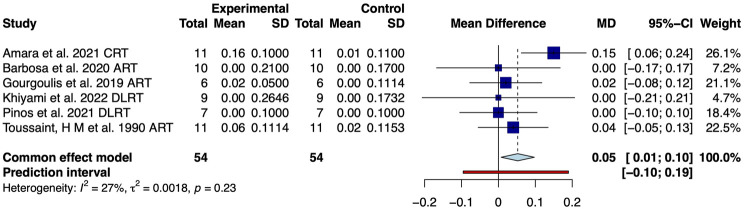
Meta-analysis of resistance training on swimming velocity in competitive swimmers.

**FIGURE 5:**
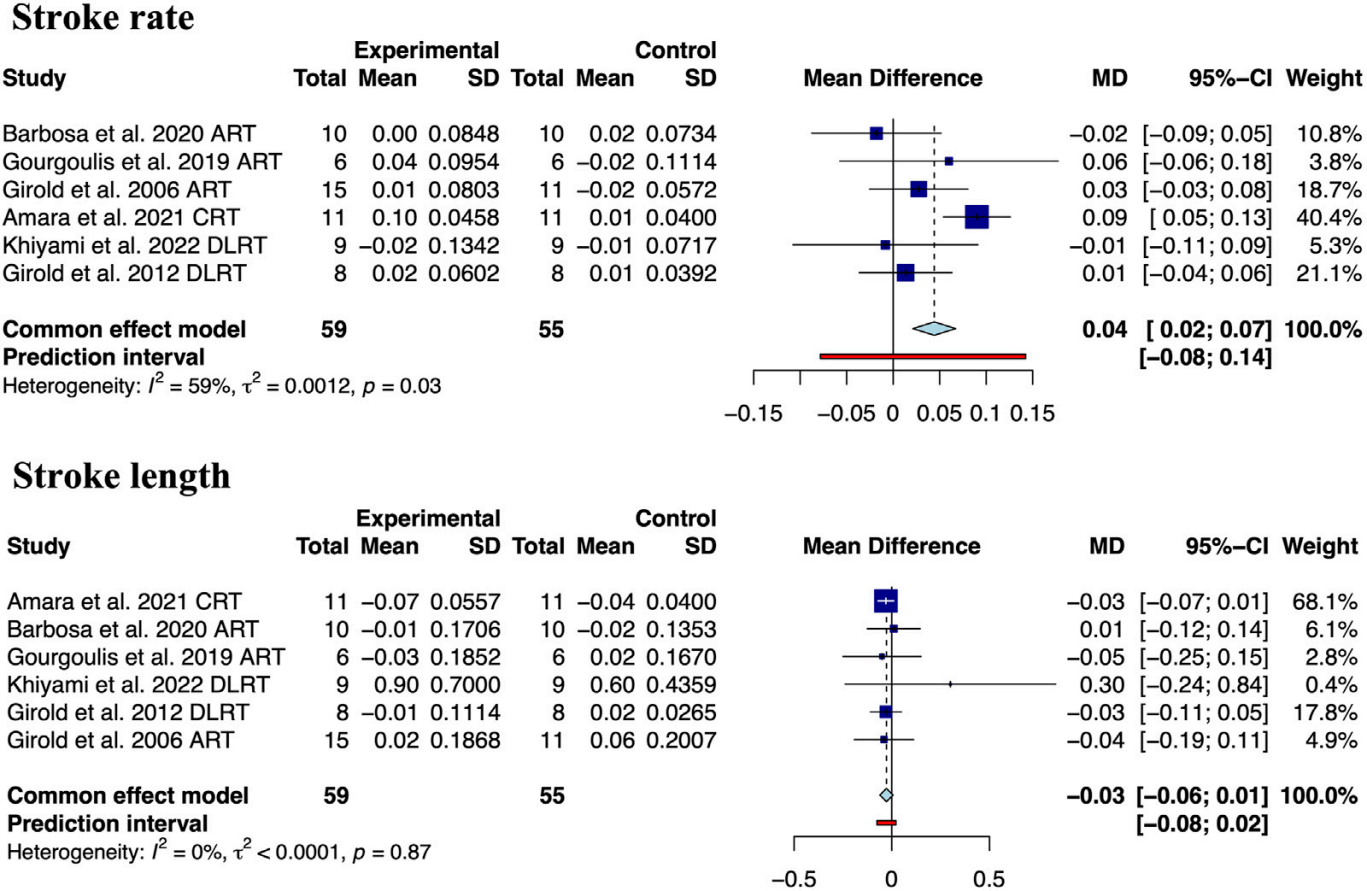
Meta-analysis of resistance training on stroke rate and stroke length in competitive swimmers.

Our results showed that resistance training significantly improves the performance of competitive swimmers in the 25 m (MD: −0.78, 95% CrI (−1.15, −0.41), I^2^ = 14.9%, 3 studies, low quality of the evidence), 50 m (MD: −0.62, 95% CrI (−1.01, −0.24), I^2^ = 0.0%, 12 studies, moderate quality of the evidence), 100 m (MD: −2.36, 95% CrI (−3.33, −1.39), I^2^ = 0.0%, 5 studies, low quality of the evidence) and 200 m front crawl (MD: −4.91, 95% CrI (−8.65, −1.18), I^2^ = 13.0%, 3 studies, very low quality of the evidence) (Figure 3). It is worth noting that the improvement in swimming performance may be due to significant improvements in velocity (MD: 0.05, 95% CrI (0.01, 0.10), I^2^ = 27.0%, 6 studies, low quality of the evidence, Figure 4) and stroke rate (MD: 0.04, 95% CrI (0.02, 0.06), I^2^ = 59.0%, 6 studies, very low quality of the evidence, Figure 5), not due to improvements in stroke length (MD: −0.03, 95% CrI (−0.06, 0.01), I^2^ = 0.0%, 6 studies, low quality of the evidence, Figure 5).

### 3.4 Subgroup analysis

The results of subgroup analysis showed that CRT (SMD: 1.60, 95% CrI (0.62, 2.59), 1 study), PT (SMD: 1.03, 95% CrI (0.25, 1.82), 1 study), and DLRT (SMD: 1.18, 95% CrI (0.53, 1.83), I^2^ = 27.7%, 2 studies) can significantly improve the maximum muscle strength of upper limbs in competitive swimmers, excepted the ART (Table 3).

**TABLE 3:** The risk of bias assessment for the individual included studies.

Study	Randomization process	Deviations from intended interventions	Missing outcome data	Measurement of the outcome	Selection of the reported result	Overall bias
Amara et al. (2022)	Low	Some concerns	Low	Low	Low	Some concerns
Amara et al. (2021a)	Some concerns	Low	Low	Low	Low	Some concerns
Barbosa et al. (2020)	Some concerns	Low	Low	Low	Low	Some concerns
Gourgoulis et al. (2019)	Low	Low	Low	Low	Low	Low
Khiyami et al. (2022)	Low	Low	Low	Low	Some concerns	Some concerns
Naczk et al. (2017)	Low	Low	Low	Low	Low	Low
Norberto et al. (2023)	Low	Low	Low	Low	Low	Low
Pinos et al. (2021)	Some concerns	Low	Low	Low	Low	Some concerns
Sammoud et al. (2021)	Low	Low	Some concerns	Low	Low	Some concerns
Sammoud et al. (2019)	Low	Low	Low	Low	Low	Low
Girold et al. (2012)	Low	Low	Low	Low	Low	Low
Girold et al. (2006)	Some concerns	Some concerns	Some concerns	Low	Low	Some concerns
Toussaint and Vervoorn (1990)	Low	Low	High	Low	Low	High

Only two forms of resistance training (CRT and PT) evaluated for their effects on the 25 m front crawl. The results of subgroup analysis showed that CRT (MD: −0.74, 95% CrI (−1.78, −0.38), 1 study), and PT (MD: −0.86, 95% CrI (−1.48, −0.24), 2 studies) can significantly improve the 25 m front crawl of competitive swimmers. Four forms of resistance training were evaluated for their effects on the 50 m and 100 m front crawl. The results of subgroup analysis showed that only CRT (50 m, MD: −1.08, 95% CrI (−1.78, −0.38), 1 study; 100 m, MD: −2.35, 95% CrI (−3.62, −1.08), 1 study) and PT (50 m, MD: −1.51, 95% CrI (−2.82, −0.19), I^2^ = 0%, 3 studies; 100 m, MD: 4.00, 95% CrI (−6.74, −1.26), 1 study) significantly improved the 50 m and 100 m front crawl respectively. Three forms of resistance training (ART, PT, and DLRT) were evaluated for their effects on the 200 m front crawl. The results of subgroup analysis showed that only PT (MD: −7.00, 95% CrI (−13.57, −0.43), 1 study) significantly improved the 200 m front crawl. Three forms of resistance training (ART, CRT, and DLRT) were evaluated for their effects on the velocity, stroke rate and stroke length. The results of subgroup analysis showed that only CRT significantly improved the velocity (MD: 0.15, 95% CrI (0.06, 0.24), 1 study) and stroke rate (MD: 0.09, 95% CrI (0.05, 0.13), 1 study), and no form of resistance training found to significantly improve stroke length.

### 3.5 Methodological quality and risk of bias assessment

Of the 13 trials, for overall bias, 5 studies were assessed as low risk of bias, 7 as some concerns and 1 as high. In the randomization process, 7 trials were low risk, 4 trials were some concerns; for deviations from intended interventions, 11 trials were at low risk, 2 trials were some concerns; in the missing outcome data, 10 trials were low risk, 2 trials were some concerns, and 1 trial was high risk; in the measurement of the outcome, 13 trials were low risk; in the selection of the reported result, 11 trials were low risk, 1 trial was some concerns (Table 4).

**TABLE 4:** Summary of subgroup analysis results of resistance training on sport performance in competitive swimmers. **Outcomes/subset:** This column lists the specific outcomes or subsets of data being analyzed in the study. **Effect size (95% CrIs):** This column reports the effect size and 95% Credible Intervals (CrIs). Effect size measures the strength of the relationship between two variables. **Egger test (*p*-value):** This column contains the p-value from Egger’s test, which is used to detect publication bias in meta-analyses. **Publication bias (no of studies):** This column indicates the presence of publication bias and the number of studies included in the analysis. **Imprecision (no of participants):** This column assesses the imprecision in the results, often related to the sample size (number of participants) in the included studies. **Heterogeneity (I^2^):** This column measures heterogeneity using the I^2^ statistic, which quantifies the percentage of variation across studies that is due to heterogeneity rather than chance. **Risk of bias:** This column evaluates the risk of bias in the included studies, assessing factors that may affect the validity of the results. **Quality of the evidence:** This column rates the overall quality of the evidence, typically using criteria like the GRADE system, to determine how confident we can be in the effect estimates.

Outcomes/subset	Effect size (95% CrIs)	Egger test (*p*-value)	Publication bias (no of studies)	Imprecision (no of participants)	Heterogeneity (I^2^)	Risk of bias	Quality of the evidence
Muscle strength	0.89 (0.54, 1.24) *	0.179	5	129	36.00%	25.0%	Low
ART	0.44 (−0.35, 1.23)	—	2	48	0.00%	—	—
CRT	1.60 (0.62, 2.59) *	—	1	22	—	—	—
PT	1.03 (0.25, 1.82) *	—	1	29	—	—	—
RT	1.18 (0.53, 1.83) *	—	2	45	27.70%	—	—
**25 ** **m (s)**	−0.78 (−1.15, −0.41) *	0.822	3	70	14.9%	0.00%	Low
CRT	−0.74 (−1.20, −0.28) *	—	1	22	—	—	—
PT	−0.86 (−1.48, −0.24) *	—	2	48	55.7%	—	—
**50 ** **m (s)**	−0.62 (−1.01, −0.24) *	0.695	12	255	0.00%	9.09%	Moderate
ART	−0.45 (−1.55, 0.66)	—	3	58	0.00%	—	—
CRT	−1.08 (−1.78, −0.38) *	—	1	22	—	—	—
PT	−1.51 (−2.82, −0.19) *	—	3	77	0.00%	—	—
RT	−0.22 (−0.78, 0.33)	—	5	91	0.00%	—	—
**100 ** **m (s)**	−2.36 (−3.33, −1.39) *	0.989	5	114	0.00%	20.00%	Low
ART	−1.03 (−4.16, 2.11)	—	2	58	0.00%	—	—
CRT	−2.35 (−3.62, −1.08) *	—	1	22	—	—	—
PT	−4.00 (−6.74, −1.26) *	—	1	29	—	—	—
RT	−2.01 (−4.17, 0.14)	—	2	43	0.00%	—	—
**200 ** **m (s)**	−4.91 (−8.65, −1.18) *	0.48	3	77	13.00%	33.00%	Very low
ART	−5.31 (−11.58, 0.96)	—	2	34	59.80%	—	—
PT	−7.00 (−13.57, −0.43) *	—	1	29	—	—	—
RT	−2.40 (−8.98, 4.16)	—	1	29	—	—	—
Velocity (m/s)	0.05 (0.01, 0.10) *	0.337	6	108	27.00%	16.67%	Low
ART	0.03 (−0.04, 0.09)	—	3	54	0.00%	—	—
CRT	0.15 (0.06, 0.24) *	—	1	22	—	—	—
RT	0.00 (−0.09, 0.09)	—	2	32	0.00%	—	—
Stroke rate (cycle/min)	0.04 (0.02, 0.06) *	0.275	6	114	59.00%	0.00%	Very low
ART	0.02 (−0.02, 0.05)	—	3	58	0.00%	—	—
CRT	0.09 (0.05, 0.13) *	—	1	22	—	—	—
RT	0.01 (−0.04, 0.05)	—	2	34	0.00%	—	—
Stroke length (m/cycle)	−0.03 (−0.06, 0.01)	0.223	6	114	0.00%	0.00%	Low
ART	−0.02 (−0.11, 0.07)	—	3	58	0.00%	—	—
CRT	−0.03 (−0.07, 0.01)	—	1	22	—	—	—
RT	−0.02 (−0.10, 0.06)	—	2	34	28.80%	—	—

ART, aquatic resistance training; CRT, concurrent resistance training RT, resistance training; PT, power training, * statistically different, *p* < 0.05.

## 4 Discussion

### 4.1 Main effects

Our results indicated large effects of resistance training on measures of maximum muscle strength of upper limbs in competitive swimmers, and significantly improved 25, 50, 100, and 200 m front crawl performance. The muscle strength in the upper limbs, main muscle groups involved in front crawl swimming propulsion (Catteau and Garoff, 1990), have great representativeness in a swimming training program’s final result (Trappe and Pearson, 1994; Maglischo and do Nascimento, 1999). Hawley et al. (1992) demonstrated a strong relationship (r = 0.82 and 0.93, respectively; *p* < 0.05) between the maximum muscle strength of upper limbs and front crawl sprint swimming performance over 25 m and 50 m. This finding was supported by Amara et al. (2022), who stated that resistance training effectively improves swimming initiation and turning performance, which was particularly important for short distance swimming. Resistance training not only improve upper body strength in competitive swimmers but may also optimize energy expenditure efficiency. According to Stian Thoresen Aspenes and Karlsen (2012), through specific resistance training, athletes could improve their propulsive efficiency in the water, which directly reduced energy expenditure during swimming. More efficient muscle use reduces the amount of energy required per stroke, allowing for higher speeds and better endurance performance during long-distance swimming. Additionally, systematic resistance training has been found to help reduce the risk of shoulder injuries, a common problem among swimmers. Previous research results showed that overuse injuries caused by repetitive shoulder movements can be effectively prevented by strengthening and stabilizing the muscles surrounding the shoulder. Strengthening these muscle groups can improve joint stability and reduce muscle strains and tendonitis that can occur during high-intensity training and competition (Tate et al., 2012; Hill et al., 2015; McKenzie et al., 2023). Together, these studies indicate that incorporating resistance training into competitive swimmers’ training programs is critical to improving their swimming performance and competitiveness.

In addition, we used specific parameters during swimming, such as swimming speed, stroke rate and stroke length, to explore how resistance training can improve the front crawl performance of competitive swimmers (time taken to test the corresponding distance). Our results showed that resistance training might have increased swimming speed and ultimately front crawl performance (time taken to test the corresponding distance) in competitive swimmers simply by increasing swimmers’ stroke rate rather than stroke length. Strass (1988) conducted low-repetition high-intensity DLRT (3 sets of 3 repetitions, 90%–100% 1RM) on 10 male competitive swimmers for a total of 24 sessions over a 6-week period, and the results showed that DLRT increased swimming speed by increasing stroke length (reducing stroke rate) and ultimately improved 25 m and 50 m front crawl swimming performance. Girold et al. (2012) conducted a randomized controlled trial of 12 sessions of low-repetition high-intensity DLRT over a 4-week period (3 sets of 6 repetitions, 80%–90% 1RM) significantly improved swimmers’ stroke length and 50 m front crawl performance, but was not significantly different from habitual aquatic training. Recently, a randomized controlled trial by Amara et al. (2021a) conducted multiple sets of high-repetition moderate-intensity CRT (3–6 sets of 6–10 repetitions, 60%–80% 1RM), the results showed that compared with habitual aquatic training, CRT did not significantly improve the stroke length of competitive swimmers, but improved the front crawl performance by increasing the stroke rate and ultimately. In addition, many randomized controlled trials of ART (multiple sets and repeated with low-intensity, increased resistance during water training through resistance bands, hand paddles, or water parachute) had not found that resistance training significantly improved the stroke length of competitive swimmers (Girold et al., 2006; Gourgoulis et al., 2019; Barbosa et al., 2020). Our review of the literature seems to suggest that higher intensity, low repetition resistance training may be responsible for improving stroke length in competitive swimmers. However, since the included literature was only RCT studies, only Girold et al. (2012) evaluated stroke length for resistance training with higher intensity and low repetitions, and the data were merged. Other studies have used resistance training with multiple repetitions at a lower intensity. Therefore, overall, resistance training did not significantly increase the stroke length of competitive swimmers, but improved swimming performance by increasing stroke rate. In addition, more high-quality studies are needed in the future to verify the effectiveness of high-intensity, low-repetition resistance training models on the stroke length of competitive swimmers.

### 4.2 Subgroup analysis

#### 4.2.1 Concurrent resistance training

A range of resistance-training interventions were considered in this review. Therefore, we performed subgroup analyzes according to different resistance training modalities. The results of our subgroup analysis showed that CRT significantly improved upper limb maximal strength, 25 m, 50 m, 100 m and key swimming parameters (swimming speed and stroke rate but not stroke length) in competitive swimmers compared with habitual aquatic training. This simultaneous integration of aquatic resistance in and dry land resistance within a periodised training programme is known as concurrent training (Amara et al., 2021a; Amara et al., 2022). The results of subgroup analysis showed that only one study reported the maximum muscle strength of the upper limbs after CRT training (Amara et al., 2021a), and compared with conventional water training, CRT significantly improved the maximum muscle strength of the upper limbs of competitive swimmers. This may be due to the similarity in the content of dry land resistance training and the 1RM bench press test. This is consistent with the training specificity principle, which states that training-related adaptations are greater when training characteristics (such as movement type, contraction pattern, and movement speed) are consistent with the test activity (Buckner et al., 2017). As mentioned above, there was a strong correlation between upper limb maximum muscle strength and front crawl swimming performance. This may be the reason why CRT significantly improves the performance of competitive swimmers (Hawley et al., 1992). In addition, another CRT study conducted both water resistance and dry lower limb resistance for 9 weeks, and the 1RM squat increased by 14.94% ± 1.32%. At the same time, the 30 m leg kick swimming performance was significantly improved (5.84% ± 0.16%) (Amara et al., 2022). This may be due to improvements in maximal lower body strength as indicated by increased 1-RM squat results. It also explained the application of effective lower limb resistance training in improving performance in swimming. What’s particularly important was that these two studies were about more than just dryland resistance training. They also used hand paddles and water parachutes to increase the resistance of water training, which may improve the transfer of dry land resistance training effects to swimming performance (Amara et al., 2022; Raineteau et al., 2023b).

#### 4.2.2 Power training

The results of our subgroup analysis showed that PT significantly improved upper limb maximal strength, as well as 25, 50, 100, and 200 m swimming performance in competitive swimmers. However, due to the lack of literature on the assessment of key parameters of swimming by PT, we were unable to assess how PT improved which key parameters of swimming and ultimately improved swimming performance. Crowley et al. (2017) systematically reviewed previous studies on the impact of PT on swimming performance of competitive swimmers. First, the authors affirmed that PT had significant benefits for competitive swimmers’ swimming performance. In addition, through a systematic analysis of previous literature, the authors found that low-volume (≤3 sets, ≤10 repetitions), high-intensity (concentric contractions as fast as possible) resistance training might reduce neuromuscular fatigue and increase strength and neuromuscular improvements, which may be the main reason for the positive impact on swimming performance (Stian Aspenes et al., 2009; Girold et al., 2012; Girold et al., 2007; Strass, 1988). It is worth noting that because the reviewed literature was not a randomized controlled trial, it was not included in this study. Two of the three studies on the effect of PT on swimming performance of competitive swimmers included in this study included 4–6 sets of training, with each set repeated 6–10 times (Sammoud et al., 2019; Sammoud et al., 2021). Another study used 2–4 sets of training, with each set repeated 4–10 times (Norberto et al., 2023). It could be seen that the intrinsic mechanism by which PT might reduce neuromuscular fatigue and ultimately improve swimming performance was not established. Research by Strass (1988) found that improvements in maximal power were more beneficial to the performance of competitive swimmers than maximal strength, and the authors suggested that this might be due to various neuromuscular adaptations. These adaptations include improved motor unit recruitment, synchronization, co-contraction, rate coding, intra- and inter-neuromuscular coordination, and neural inhibition. Thus, improvements in power were effectively transferred to swimming performance, resulting in a significant increase in speed (*p* < 0.001). This could be the main reason why power training (PT) significantly improved the swimming performance of competitive swimmers.

#### 4.2.3 Dry-land resistance training

Our research results showed that compared with habitual aquatic training, DLRT only significantly improved the upper limb maximum muscle strength of competitive swimmers but did not significantly improve swimming performance and key technical parameters. This was consistent with previous research results (Tanaka et al., 1993; Song et al., 2009). Similarly, the study by Crowley et al. (2017) gave the reason that it was possible that fatigue caused by higher training volume caused DLRT to not effectively improve the swimming performance of competitive swimmers. Previous research results had shown that 8 weeks of DLRT, 3 times/week, 3 sets of 8–12 repetitions, did not cause a significant increase in serum cortisol, an indicator of overtraining (Tanaka et al., 1993). In addition, recent studies had compared the effects of different DLRT training volumes on the swimming performance of competitive swimmers, and the results had shown that high (5-6 sets, 3–5 repetitions, 85%–95% 1RM), moderate (4-5 sets, 3–5 repetitions, 85%–95%1RM) and low (3-4 groups, 3–5 repetitions, 85%–95% 1RM) training volume groups showed similar improvements in swimming performance compared with the pretest, and it did not change due to the difference between the groups caused by high and low training volumes (Amara et al., 2021b). Clearly, the level of DLRT training volume and whether it would cause fatigue were not the reasons why DLRT did not significantly improve the swimming performance of competitive swimmers. However, unfortunately, this study had not set up a habitual aquatic training, and we could not know whether DLRT with different training volumes could significantly improve the swimming performance of competitive swimmers compared with the habitual aquatic training. It was for this reason that we did not include this study. More high-quality studies might have been needed in the future to further explore the differences between DLRT and the habitual aquatic training in improving swimming performance in competitive swimmers.

#### 4.2.4 Aquatic resistance training

It was worth noting that only ART, which increased resistance during water training through resistance bands, hand paddles, or water parachute, did not significantly increase maximum muscle strength of upper limbs, the swimming performance and key technical parameters in competitive swimmers compared with the habitual aquatic training. This finding raised important questions about existing swimming training models and triggers further research into effective training methodologies. Previous research results showed that tethered swimming (ART) showed to be similar to front-crawl swimming in terms of muscle activation (Bollenes, 1988) and propulsive forces (r = 0.92, *p* < 0.01) (Morouço et al., 2011). Therefore, for competitive swimming, coaches are keen to increase resistance through resistance bands, hand paddles, or water parachute during training to improve the performance of competitive swimmers. Augusto C. Barbosa et al. (2020) conducted a study on 20 competitive swimmers over a 4-week period, totaling 12 sessions of ART (with hand paddles). The results indicated that compared to baseline and the habitual aquatic training, ART did not significantly improve front-crawl swimming performance. When the training sessions increased to more than 30, several studies proved that ART effectively enhanced the front-crawl swimming performance and upper limb muscle strength of competitive swimmers compared to before the training. However, it is important to note that this improvement was not significantly greater than that of the habitual aquatic training groups (Toussaint and Vervoorn, 1990; Girold et al., 2006; Gourgoulis et al., 2019). Therefore, we speculate that simply increasing resistance through resistance bands, hand paddles, or water parachutes during in-water training does not provide an additional improvement in the athletic performance of competitive swimmers compared to the habitual aquatic training groups. On one hand, this methodology of resistance might be too small to provide sufficient stimulus. On the other hand, due to the acceptance by coaches, this training modality (ART) might have become normalized. Thus, competitive swimmers might have already exhibited adaptation effects.

### 4.3 Limitation

Our study had several limitations. First, the literature we included focused solely on the impact of various resistance training on competitive swimmers’ front-crawl swimming performance, and these results cannot be transferred to other swimming strokes. Therefore, our findings lack universality. Second, the small amount of literature included led to specific resistance training methodology having only one study in our subgroup analysis, which was the main reason for the low credibility of our results. In addition, the studies included in this meta-analysis involved a wide range of ages and swimming training years, which may have an impact on the interpretation and generalizability of the study results. Age and years of training are important factors that affect swimming performance and training adaptability. Different ages and main events of the swimmer may have significant differences in physiology, biomechanics, and psychological status, and these differences may affect training effects and competition performance (Gonjo et al., 2020; Gonjo et al., 2021; Raineteau et al., 2023a). Likewise, length of swimming experience may also have a decisive impact on an athlete’s performance. Therefore, differences in these factors may limit the generalizability of our analytical results, and future studies should consider the potential effects of these variables and attempt more detailed subgroup analyses. Due to the limited number of studies, we were unable to conduct an in-depth analysis of the dosage of specific resistance training. Previous literature has shown that specific dosages (training periods, sets, repetitions, and intensity) significantly affect the effect of resistance training on athletic performance (Lesinski et al., 2016). Therefore, future research should involve a large number of high-quality studies to thoroughly explore the impact of resistance training dosage on competitive swimmers’ performance, providing clear training guidelines for coaches and competitive swimmers. Lastly, in this study, we did not conduct a detailed distinction and analysis of the differences between men and women in the effectiveness of training administration. This also may be a limitation of this study, and future research can further explore the impact of gender factors on training effects.

## 5 Conclusion

Resistance training is highly effective for competitive swimmers in enhancing upper limb maximal strength and front-crawl swimming performance, with significant improvements in performance potentially arising solely from increased stroke rate induced by resistance training, rather than stroke length. It is important to note that the effects of resistance training can vary depending on the methodology of training. For example, both CRT and PT are effective forms of resistance training for improving upper limb maximal strength, front-crawl swimming performance, and key swimming technical parameters. However, simply increasing resistance during swimming (ART) is not an effective training modality. Additionally, DLTRT only improves the maximal muscle strength of the upper limbs in competitive swimmers, and this effect does not translate into swimming performance.

## Data Availability

The original contributions presented in the study are included in the article/Supplementary Material, further inquiries can be directed to the corresponding author.

## Abbreviations

RCTs, Randomized Controlled Trials; SDs,standard deviations; SEs, standard errors; CrIs, credible intervals; MD, mean differences; SMD, standard mean difference; CRT, concurrent resistance training; ART, aquatic resistance training; DLRT, dry-land resistance training; PT, power training; RT, resistance training; HAT, habitual aquatic training; NA, non-available.
